# Concentrations of Plasma Nucleosomes but Not Cell-Free DNA Are Prognostic in Dogs Following Trauma

**DOI:** 10.3389/fvets.2018.00180

**Published:** 2018-07-30

**Authors:** Jo-Annie Letendre, Robert Goggs

**Affiliations:** ^1^Centre DMV, Montreal, QC, Canada; ^2^Department of Clinical Sciences, College of Veterinary Medicine, Cornell University, Ithaca, NY, United States

**Keywords:** injury, cfDNA, biomarkers, NETs, NETosis, ATT, APPLE, survival

## Abstract

Trauma is common in dogs and causes significant morbidity and mortality, but it remains a challenge to assess prognosis in these patients. This study aimed to investigate the use of plasma cell-free DNA (cfDNA) and nucleosome concentrations as prognostic biomarkers in canine trauma. Using a prospective, observational case-control study design, 49 dogs with trauma were consecutively enrolled from 07/2015 to 10/2017 and followed to hospital discharge. Dogs with animal trauma triage (ATT) scores ≥3 at presentation were eligible for enrollment. Dogs <3 kg or with pre-existing coagulopathies were excluded. Thirty-three healthy control dogs were also enrolled. Illness and injury severity scores were calculated using at-presentation data. Plasma cfDNA was measured in triplicate using a benchtop fluorimeter. Plasma nucleosome concentrations were determined in duplicate by ELISA. Mann-Whitney *U* tests were used to compare biomarker concentrations between groups and between survivors and non-survivors. Associations between biomarkers were evaluated using Spearman's correlation coefficients. Alpha was set at 0.05. Concentrations of cfDNA and nucleosomes were significantly higher in injured dogs compared to healthy controls (*P* ≤ 0.0001). Nucleosomes and cfDNA concentrations were positively correlated (*r*_s_ 0.475, *P* < 0.001). Concentrations of both cfDNA and nucleosomes were correlated with shock index (*r*_s_ 0.367, *P* = 0.010, *r*_s_ 0.358, *P* = 0.012 respectively), but only nucleosomes were correlated with ATT (*r*_s_ 0.327, *P* = 0.022) and acute patient physiology and laboratory evaluation (APPLE) scores (*r*_s_ 0.356, *P* = 0.012). Median nucleosome concentrations were significantly higher in non-survivors than in survivors [8.2 AU (3.1–26.4) vs. 1.6 AU (0.5–5.2); *P* = 0.01]. Among illness severity scores, only APPLE was discriminant for survival (AUROC 0.912, *P* < 0.001). In summary, in moderately-severely injured dogs, high nucleosome concentrations are significantly associated with non-survival.

## Introduction

Dogs are frequently presented to veterinary emergency rooms following trauma, and the injuries resulting from blunt force trauma and animal bites are associated with considerable morbidity and mortality (1). Dogs that survive the initial trauma and receive timely intensive care have a better prognosis for survival, while development of complications such as pneumonia, disseminated intravascular coagulation or multiple organ failure increases the risk of death in dogs followinginjury (2).

It is challenging for clinicians to predict the development of complications or assess the prognosis of traumatized dogs. Systems such as the Animal Trauma Triage (ATT) score and the canine acute patient physiologic and laboratory evaluation (APPLE) score were developed to aid the initial evaluation of such patients (3, 4). Early and repeated patient scoring can help guide therapeutic interventions and may aid assessment of prognosis (5–7). While these methods aid prognostication of hospitalized patients, they provide limited biologic insight into the pathophysiologic effects of tissue injury and shock. Biomarkers have been widely evaluated in human trauma in an attempt to provide accurate diagnosis and prognostication (8–11), and are integral to the ongoing pursuit of individualized medicine (12). Identification of an accurate, easy to measure bedside biomarker that correlates with injury severity and prognosis in injured dogs would be very valuable.

Increased plasma concentrations of cell-free DNA (cfDNA) are detectable in people following trauma and are associated with mortality (13). The source of this cfDNA is uncertain, but it may originate from neutrophil extracellular traps (NETs), apoptotic cells or necrotic tissue (14, 15). NETs are a host-defense mechanism, comprised of extracellular DNA decorated with histones and bactericidal proteins including elastase and myeloperoxidase, produced by activated neutrophils that ensnare, trap and opsonize invading bacteria (16). In people, it has been shown that plasma cfDNA concentrations increase early after injury (17), and remain increased in more severely injured patients (18). Persistently increased cfDNA concentrations correlate with mortality in traumatic brain injury patients and increased cfDNA concentrations are related to the development of posttraumatic complications in blunt force trauma patients (19). Recently, it was reported that plasma cfDNA concentrations are also increased in dogs following trauma (20).

Nucleosomes are complexes formed by DNA and histone proteins that are released into circulation during cell death and cellular damage such as apoptosis and necrosis (21). They can also be due to the process of NET formation (22). As such, cfDNA and nucleosomes share potential origins, but are distinct entities (23), with differential potential for immune cell activation through pattern recognition receptors (21). Measuring both biomarkers may provide better insights into the disease process than either alone. NET formation may directly contribute to pathogenesis of trauma through the phenomenon of immunothrombosis (24), and both nucleosomes and cfDNA may contribute to the pathogenesis of the acute coagulopathy of trauma-shock (25, 26). Humans and dogs with sepsis have significantly increased plasma nucleosome concentrations (27, 28), and nucleosomes are increased in human trauma (29), but to the authors' knowledge, nucleosome concentrations in canine trauma have not been evaluated to date.

Therefore, the purpose of the present study was to investigate the utility of plasma cfDNA and nucleosomes as prognostic biomarkers in canine trauma and to determine if plasma cfDNA was associated with plasma nucleosome concentrations. The study also aimed to evaluate the associations between illness and injury severity scores and survival to hospital discharge. It was hypothesized that in dogs following trauma, plasma cfDNA and nucleosome concentrations are higher than in healthy dogs, are positively correlated with injury severity and with each other, and are higher in non-survivors. It was also hypothesized that illness and injury severity scores are associated with survival to discharge in dogs following trauma.

## Materials and methods

### Animals

A priori sample size calculations were performed with an online calculator (SISA-Sample Size, Quantitative Skills http://www.quantitativeskills.com/sisa/calculations/samsize.htm), using mortality estimates from published studies (2, 4). The study aimed to identify the difference between the 25th and the 75th percentiles in the pilot cfDNA data. It was estimated that 42 dogs at 14% mortality would enable detection of a significant difference between these percentiles. It was intended that an additional 6 dogs (15%) would be enrolled to allow for withdrawal or loss to follow-up. Blood samples were collected at hospital admission from 49 dogs following moderate-severe trauma (ATT ≥3) enrolled between 07/2015 and 10/2017. Additional details on other biomarkers measured in samples from the dogs described in this study are presented in an accompanying manuscript (30).

Blood samples were collected at the time of intravenous catheter placement or at the time of venipuncture for collection of blood samples for clinician directed point-of-care testing prior to initiation of therapy. Dogs weighing less than 3 kg and those with a known, pre-existing coagulopathy were excluded to minimize risks associated with blood sampling. Respective primary clinicians determined all aspects of patient management but were not made aware of patient biomarker concentrations. Apart from blood samples collected as part of the present study, all other diagnostic testing was at the discretion of the attending clinician.

For the injured dog population, the primary outcome measure was death or euthanasia for disease severity prior to hospital discharge. Specifically, patients were euthanized due to disease severity when the attending clinician advised clients that a dog's injuries were not felt to be survivable, or their condition was acutely deteriorating despite therapy, or the prognosis for survival and return to function was judged to be grave. No dogs were euthanized solely for financial limitations. Study participation was undertaken with written informed client consent under local IACUC approval (2014-0053). A population of healthy dogs was also recruited, also with written informed consent under local IACUC approval (2014-0052). Arbitrarily, the intention was to recruit 2 controls for every 3 cases, but one additional control dog was enrolled over this intended number (total *n* = 33). Healthy controls dogs were eligible if they had no history or evidence of recent or chronic medical conditions and had not received any medication, except for routine preventative healthcare, within the preceding 3 months. Dogs were classified healthy on the basis of history, physical examinations, and complete blood count, and serum chemistry results.

### Data collection

Signalment, previous medical history and physical examination findings at presentation were recorded. Blood pressure was measured non-invasively by oscillometric (Cardell 9401, Midmark, Dayton, OH) or Doppler methods. Blood hemoglobin oxygen saturation was measured by pulse oximetry (Rad-87, Masimo, Irvine, CA). Mentation, modified Glasgow coma scale (31) and ultrasound body cavity fluid scores were assessed at admission to enable calculation of the Acute Patient Physiologic and Laboratory Evaluation (APPLE and APPLE_fast_) scores (6). Where missing data needed for illness severity scoring were encountered, the median value for the remainder of the population was used instead (median imputation) similar to that performed during derivation of the APPLE score (6). The shock index, sequential organ failure assessment (SOFA) and survival prediction index-2 (SPI2) scores were calculated as previously reported (5, 7, 32). Blood samples were collected at enrollment for blood lactate (Lactate Pro, Arkray, Edina, MN), complete blood count (ADVIA 2120, Siemens, Washington, DC), serum biochemistry profiles (Modular P, Roche-Hitachi, Indianapolis, IN), and cfDNA measurement. The remainder of the citrated plasma was frozen at −80°C for batched analysis of plasma nucleosomes. The maximum duration of storage before analysis was 15 months.

Measurement of cfDNA was conducted as follows. Citrated plasma was generated by centrifugation of whole blood for 10 min at 1,370 g (Ultra-8V Centrifuge, LW Scientific, Lawrenceville, GA). After centrifugation, plasma was decanted into polypropylene freezer tubes (Polypropylene Screw-Cap Microcentrifuge Tubes, VWR, Radnor, PA), by pipetting. Some plasma was deliberately left in the tube to minimize the risk of disturbing the buffy coat. Concentrations of cfDNA in citrated plasma were measured immediately following sample collection using a benchtop fluorimeter and relevant reagents (Qubit 3.0 Fluorometer and Quant-It HS dsDNA Kit, Life Technologies, Waltham, MA), according to the manufacturers' instructions (33, 34). Concentrations of plasma nucleosomes were analyzed in 2 batches using a commercial ELISA (Cell Death Detection ELISA Plus, Roche, Indianapolis, IN) scaled against pooled normal canine plasma (35). Pooled plasma was employed to provide a normal value because the ELISA lacks a reference standard. Plasma pooled from multiple normal dogs was analyzed in four wells on each plate and a reference value established as the mean of these four replicates. This reference value was assigned an arbitrary unit (AU) value of 1.0. Nucleosome concentrations for all other patients were expressed relative to that value.

### Statistical methods

Prior to test selection, data were assessed for normality by assessment of histograms, calculation of skewness and kurtosis and with the D'Agostino Pearson test and descriptive statistics calculated. Most variables were not normally distributed and hence all are reported as median (IQR). Continuous variables were compared between groups (e.g., controls vs. trauma cases, survivors vs. non-survivors) with the Mann-Whitney U test. Associations between cfDNA and plasma nucleosomes and between these biomarker concentrations and vital signs, illness severity scores and patient parameters were evaluated with scatterplots and by calculation of Spearman's correlation coefficients (r_s_). For categorical variables, 2 × 2 contingency tables were constructed to compare frequencies using Fisher's exact test and calculation of odds ratios with 95% confidence intervals.

Values associated with non-survival to hospital discharge were evaluated by plotting receiver-operating characteristic (ROC) curves and through calculation of the area-under these curves (AUROC). These analyses were exploratory and were not intended to validate these scores. Multivariable logistic regression was used to determine if cfDNA or nucleosomes were associated with survival to hospital discharge independent of disease severity based on the APPLE score. Candidate variables identified with univariate analyses were entered using a forward stepwise method, using *P* < 0.05 for the likelihood ratio to add explanatory variables to the model. Model accuracy was determined using 2 × 2 classification tables. Model discrimination was determined by calculating AUROC. Model calibration was assessed by Hosmer–Lemeshow goodness-of-fit (model rejected if *P* < 0.05) and visual inspection of contingency tables. Model utility was assessed using Nagelkerke's R^2^. The optimal cut off for sensitivity and specificity was identified by maximizing the Youden index (J) where J = (Sensitivity+Specificity)-1 (36). All analyses were performed using commercial software (Prism 6.0, GraphPad, La Jolla, CA; SPSS Statistics 24, IBM, Armonk, NY). Alpha was set at 0.05.

## Results

### Demographics

The study enrolled 49 dogs following trauma, and 33 healthy control dogs. Among the injured dogs, there were 16 mixed breed dogs, 6 Labrador retrievers, 3 German shepherd dogs, 2 border collies, 2 German short-haired pointers, 2 Rhodesian ridgebacks and 2 Staffordshire bull terriers. There were 16 other purebred dogs, all *n* = 1. There were 16 male neutered dogs, 9 male intact dogs, 16 female spayed dogs and 8 female intact dogs. The median age was 4 years (1.15–7.75) and the median bodyweight was 24.0 kg (10.0–35.0). The healthy dogs consisted of 18 mixed breed dogs, 4 Labrador retrievers, 2 German short-haired pointers, 2 Golden retrievers, 2 Staffordshire bull terriers, and 4 other pure breed dogs. There were 12 male neutered dogs, 5 male intact dogs, 15 female spayed dogs and 1 female intact dogs. The median age was 3 years (1.95–5.25) and the median bodyweight was 23.6 kg (15.1–31.2). Age, weight, and proportion of males vs. females was not significantly different between the trauma group and the controls.

In the trauma dog population, the estimated median time interval between injury and presentation was 2.5 h (1–5). The mechanism of injury was blunt force trauma (struck by vehicle) in 81.6% (40/49), penetrating trauma (dog bites) in 14.2% (7/49), crush injury (*n* = 1) and fall from moving vehicle (*n* = 1). Following injury, 46.9% (23/49) were assessed by a primary care veterinarian before being referred to the study institution. The other 26 dogs (53.1%) presented directly to the study hospital. Of the 23 dogs seen by a primary care veterinarian prior to referral, 18 dogs received 43 treatments in total, median 2 (1–3). Treatments included opioid analgesia (*n* = 11), fluid therapy (*n* = 9), antimicrobials (*n* = 6), glucocorticoids (*n* = 5), hyperosmolar agents (*n* = 3), unspecified analgesics (*n* = 3), alpha-2-agonists (*n* = 2), non-steroidal anti-inflammatory drugs (*n* = 2), antiemetics (*n* = 1), gastroprotectants (*n* = 1). Patient vital parameters, injury severity scores and the results of initial point-of-care assessments recorded at presentation to the study institution are summarized in Table 1. Of these parameters, only temperature was significantly different between dogs that were referred 100.3°F (98.8–101.1) [37.9°C (37.1–38.4)] compared to those that presented primarily to the study institution 101.3°F (100.5–101.8) [38.5°C (38.1–38.8)] (*P* = 0.0095). There were 3 dogs that did not have a complete blood count or a serum chemistry panel available. For these patients, the median values for albumin, creatinine and total bilirubin (*n* = 46) were used to enable APPLE score calculation and the median platelet count (*n* = 46) was used to enable APPLE_fast_ score calculation. There were 3 dogs where the only blood pressure reading available was obtained by Doppler methodology. For these 3 patients median imputation of mean arterial pressure values was used for SPI2 score calculation.

**Table 1:** Summary statistics for patient vital parameters, injury severity scores and the results of initial point-of-care assessments recorded at presentation to the study institution.

	***n***	**Mean**	**SD**	**Min**	**25th%**	**Median**	**75th%**	**Max**
T (°F)^*^	49	100.6	1.4	97.5	99.7	100.9	101.5	103
T (°C)	49	36.9	6	8.2	37.5	38.2	38.6	39.4
PR^*^	49	138	38	60	111	140	163	220
RR	49	42	21	12	28	36	51	100
SpO_2_	48	95	5	74	94	96	98	100
SAP	49	139	33	78	116.5	139	159.5	250
MAP^*†*^	46	103	24	61	86	100	118	177
Lactate	49	3	1.8	0	1.7	2.7	3.6	7.9
Shock index	49	1.07	0.46	0.34	0.78	1.02	1.35	2.58
Fluid score	49	NA	NA	0	0	0	1	1
MGCS	49	NA	NA	7	15	16	17	18
Mentation	49	NA	NA	0	1	2	3	4
ATT	49	NA	NA	3	4	5	6	13
SPI2	49	NA	NA	0.66	1.65	2.19	2.63	3.67
APPLE	49	NA	NA	6	20	26	33	49
APPLE_fast_	49	NA	NA	12	19	23	28	40
SOFA	49	NA	NA	0	0.5	1	2	6

* *These parameters were normally distributed; the remainder were non-parametric*.

† *For the MAP, only 46 values were available, because 3 patients had only a Doppler blood pressure recorded. Doppler blood pressure readings were included in the SAP summary statistics. All other parameters were non-parametric. APPLE, acute patient physiologic and laboratory evaluation; ATT, acute trauma triage score; MAP, mean arterial pressure; PR, pulse rate; RR, respiratory rate; SAP; systolic arterial pressure; SOFA, sequential organ failure assessment score; SPI2, survival prediction index-2; T, temperature*.

### Patterns of injury

Within the 49 injured dogs, 164 separate injuries were described. Skin wounds (including bite wounds, lacerations, degloving injuries and abrasions) were the most frequently reported (*n* = 25). Fractures were also common, affecting the appendicular skeleton (*n* = 19), pelvis (*n* = 18), spine (*n* = 8), facial bones (*n* = 7), dentition (*n* = 5), ribs (*n* = 4), hard palate (*n* = 1), hyoid apparatus (*n* = 1), cranial vault (*n* = 1) and scapula (*n* = 1), while luxations of the sacroiliac (*n* = 6), coxofemoral (*n* = 5), elbow (*n* = 1), and glenohumeral (*n* = 1) joints were also reported. Other injuries included hemoabdomen (*n* = 13), pneumothorax (*n* = 12), pulmonary contusions (*n* = 11), head trauma (*n* = 9), surgically confirmed abdominal visceral injury (*n* = 4), diaphragmatic rupture/hernia (*n* = 2), hemothorax (*n* = 2), pneumomediastinum (*n* = 2), retroperitoneal hemorrhage (*n* = 2), arrhythmia (*n* = 1), brachial plexus avulsion (*n* = 1), collateral ligament rupture (*n* = 1), and globe rupture (*n* = 1).

### Outcome

**Figure 1:**
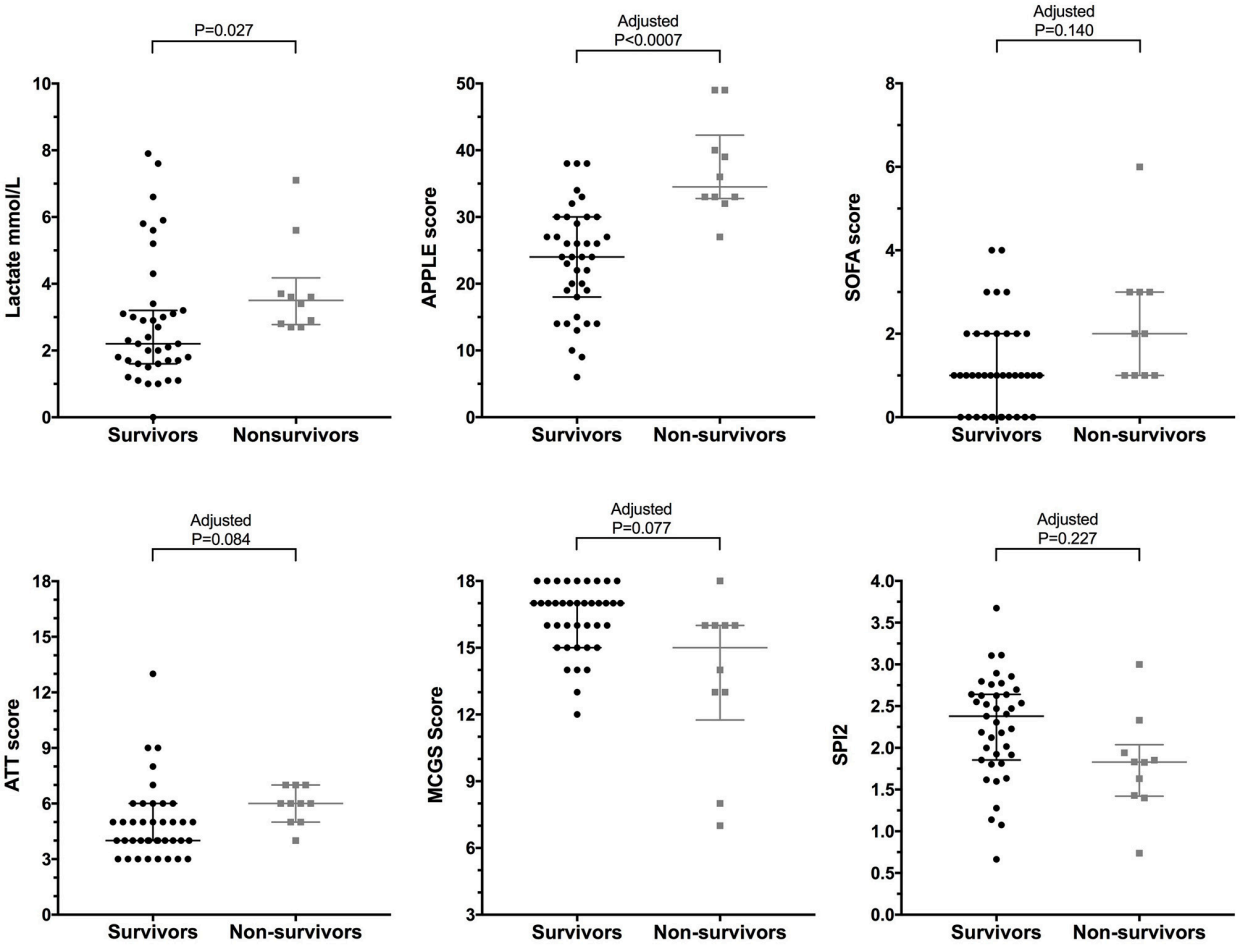
Scatterplots of lactate and illness severity scores from dogs following moderate-severe trauma grouped as survivors (•) (*n* = 39) vs. non-survivors (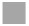) (*n* = 10). The central horizontal line represents the median, and the two error bars represent the interquartile range. Comparisons between the groups were performed using the Mann-Whitney U test. *P* < 0.05 was considered significant. *P*-values for illness severity score comparisons were adjusted using the Bonferroni correction (*n* = 7 comparisons), as indicated.

**Figure 2:**
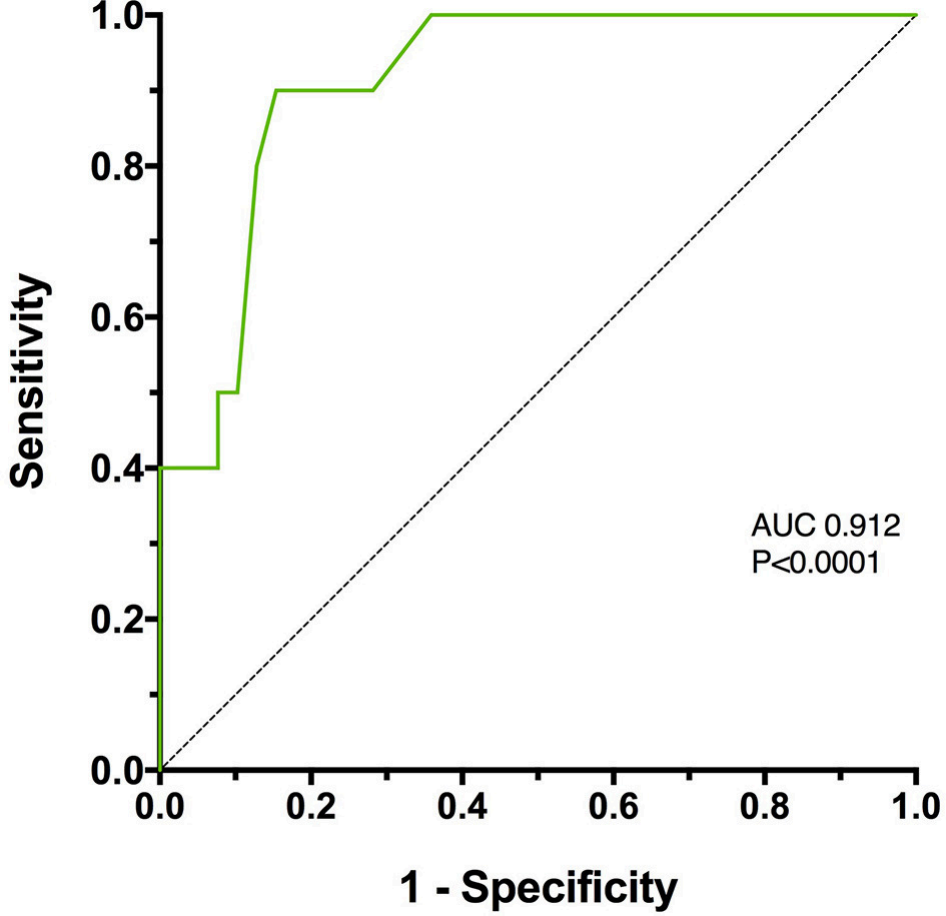
A receiver operating characteristic (ROC) curve of Acute Patient Physiology and Laboratory Evaluation (APPLE) score assessing its association with survival in 49 dogs following moderate-severe trauma. The area under the curve (AUC) was 0.912, which was significantly greater than 0.5 (*P* < 0.0001).

The mortality rate for the injured dogs was 20.4% (10/49) of which 9 (90%) were euthanized and 1 (10%) died naturally. No dogs were euthanized for financial limitations; all dogs were euthanized due to injury severity. Of the 10 dogs that were euthanized, 7 were euthanized prior to hospitalization (i.e., within hours of presentation). Among the 3 hospitalized non-survivors, 2 dogs were euthanized and 1 dog died; none of these dogs survived more than 1 day. Among the survivors the median duration of hospitalization was 4 days (2–5) prior to discharge. Two dogs were discharged against medical advice (these dogs were excluded from the duration of hospitalization calculation). A significantly greater proportion of dogs that presented primarily to the study institution died or were euthanized (34.6%), compared to those that were referred (4.3%) (OR 11.65, 95% CI 1.76–132.70, *P* = 0.012). There was no significant difference in the frequency of death or euthanasia in dogs with blunt force trauma (struck by vehicle) compared to other mechanisms (dog bites, crush injuries, and fall from moving vehicle) (*P* = 0.663). After the Bonferroni correction for multiple comparisons was applied to the illness severity scores, only lactate and APPLE were significantly different between survivors and non-survivors (Figure 1, Table 2). The most discriminant prognostic marker was the APPLE score. Generation of ROC curves indicated that the APPLE score was the most discriminant for outcome (AUROC 0.912, *P* < 0.001). Calculation of the Youden index suggested that at an optimal cut-off for the APPLE score was 31, which was 90% sensitive and 84.6% specific for non-survival (Figure 2). Sensitivity and specificity values with optimal cut-offs for the illness severity scores are detailed in Table 2.

**Table 2:** Patient vital parameters, injury severity scores and the results of initial point-of-care assessments recorded at presentation to the study institution—survivors vs. non-survivors.

	**Survivors (*n* = 39)**		**Non-survivors (*n* = 10)**						
	**Median**	**IQR**	**Median**	**IQR**	***P***	**Adjusted P**	**AUROC (95% CI)**	**Sens**	**Spec**
SAP (mmHg)	141	117–158	129	93–168	0.459	–	0.587 (0.348–0.808)	60.0%	66.7%
MAP^*†*^ (mmHg)	103	91–118	96.5	81–101	0.225	–	0.627 (0.448–0.805)	80.0%	53.9%
Lactate (mmol/L)	2.2	1.6–3.2	3.5	2.8–4.2	**0.027**	–	0.733 (0.595–0.872)	100%	56.4%
Shock index (AU)	1.02	0.78–1.27	1.16	0.77–1.52	0.426	1.000	0.585 (0.363–0.806)	50.0%	76.9%
MGCS (AU)	17	15–17	15	12–16	0.011	0.077	0.756 (0.577–0.936)	90.0%	56.4%
ATT (AU)	4	4–6	6	5–7	0.012	0.084	0.750 (0.604–0.896)	70.0%	74.4%
SPI2 (AU)	2.38	1.85–2.64	1.83	1.42–2.04	0.032	0.227	0.721 (0.542–0.899)	80.0%	69.2%
APPLE (AU)	24	18–30	35	33–42	**<0.0001**	**<0.0007**	0.912 (0.878–0.995)	90.0%	84.6%
APPLE_fast_ (AU)	23	18–26	28	20–34	0.081	0.567	0.681 (0.486–0.876)	50.0%	84.6%
SOFA (AU)	1	0–2	2	1–3	0.020	0.140	0.730 (0.570–0.889)	100%	30.8%

*P-values in bold type were significant at P < 0.05. For illness severity scores, a Bonferroni correction was applied to adjust for multiple comparisons (n = 7)*.

† *For the MAP data, n = 37 in the survivors group and 9 in the non-survivors group because for 3 dogs only a Doppler blood pressure was available. Those data are included in the SAP statistics. APPLE, acute patient physiologic and laboratory evaluation; ATT, acute trauma triage score; MAP, mean arterial pressure; SAP; systolic arterial pressure; SOFA, sequential organ failure assessment score; SPI2, survival prediction index-2*.

### Point-of-care cfDNA

**Figure 3:**
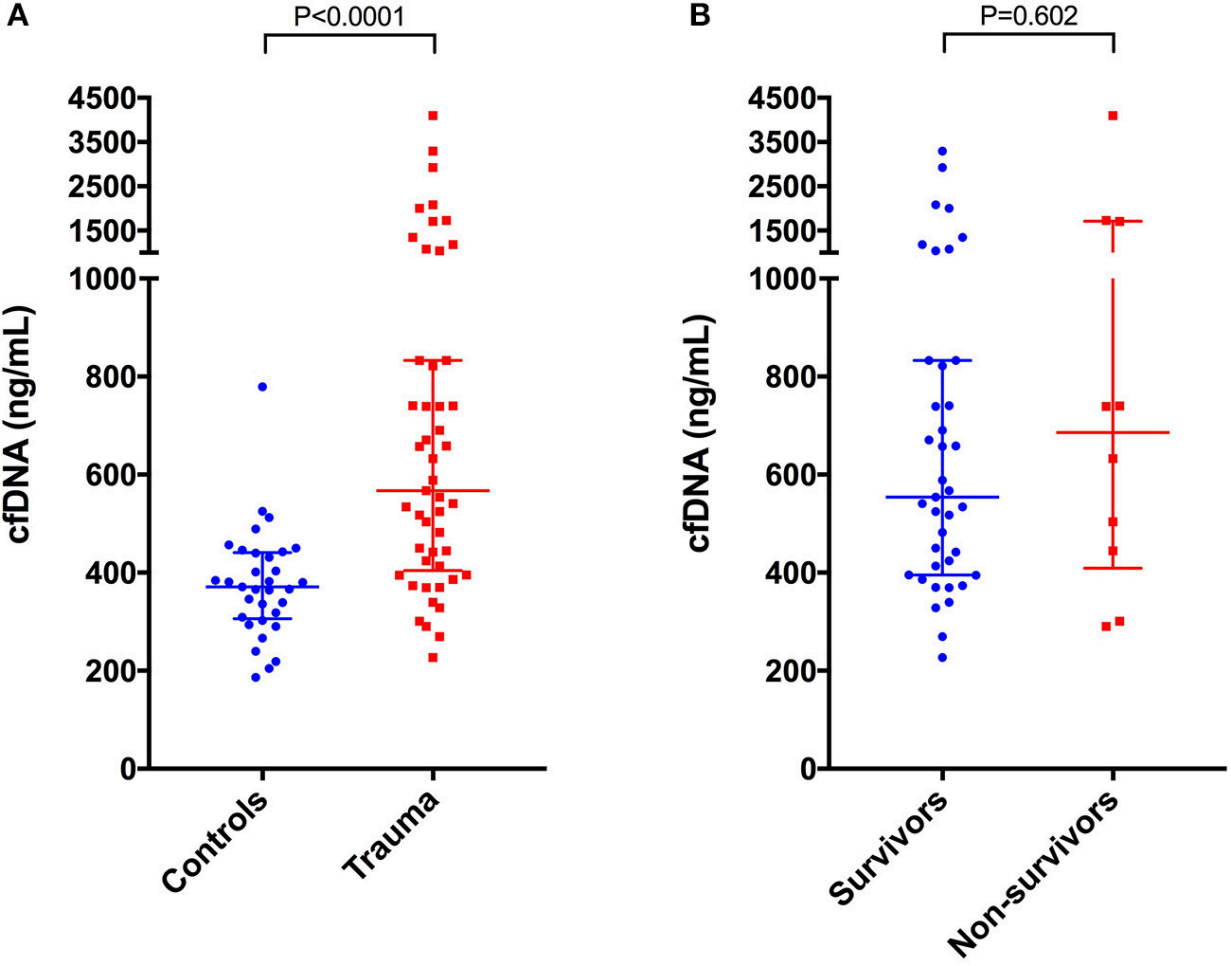
Scatterplots of plasma cell-free DNA (cfDNA) in **(A)** healthy controls (*n* = 33) compared to dogs following moderate-severe trauma (*n* = 49), and in **(B)** survivors (*n* = 39) compared to non-survivors (*n* = 10). The central horizontal line represents the median, and the two error bars represent the interquartile range. Comparisons between the groups were performed using the Mann-Whitney U test. *P* < 0.05 was considered significant.

**Figure 4:**
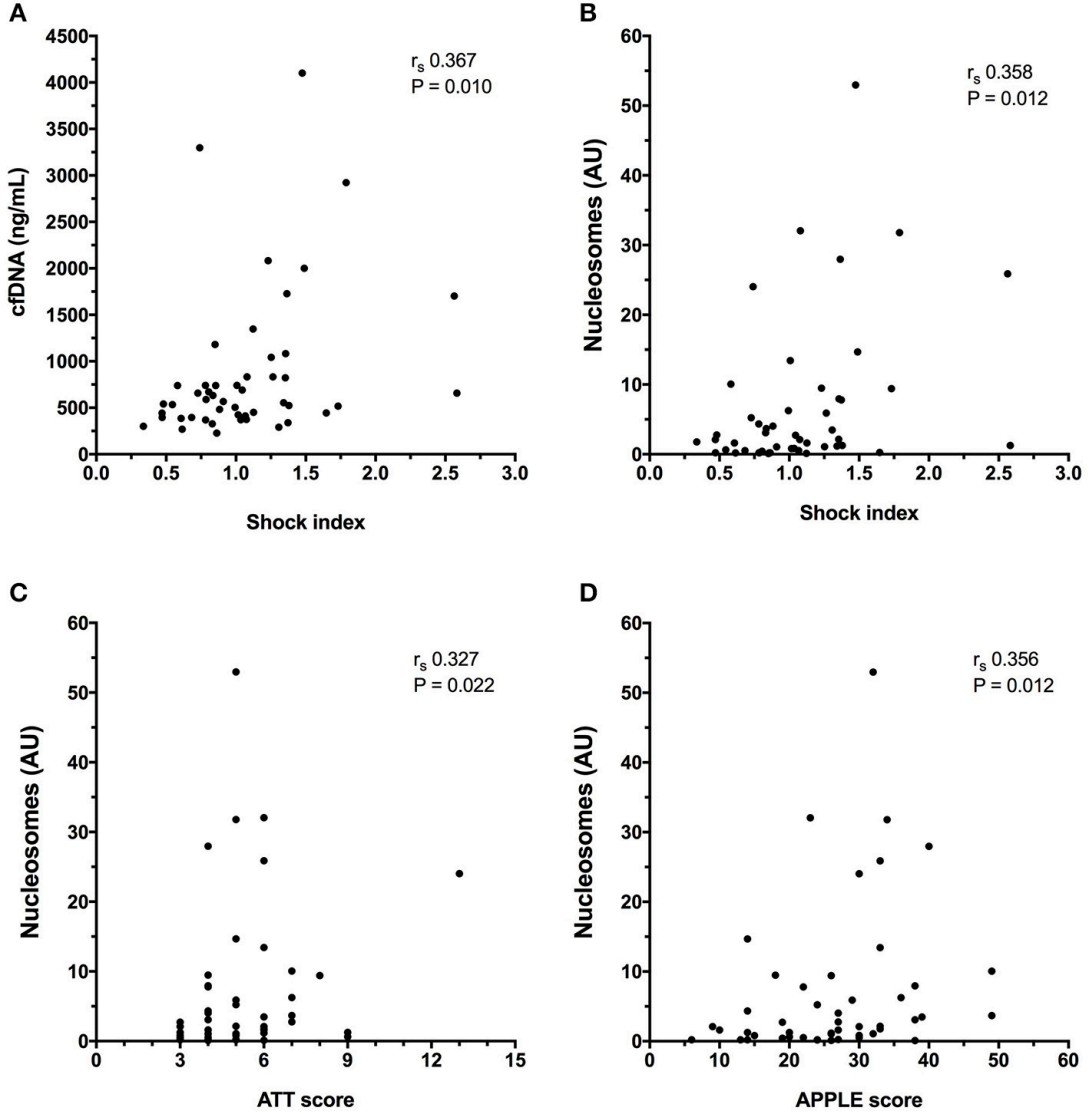
X-Y scatterplots of illness severity scores vs. biomarker concentrations. **(A)** Shock index vs. plasma cell-free DNA (cfDNA) concentration, **(B)** Shock index vs. plasma nucleosome concentration in arbitrary units (AU), **(C)** Animal Trauma Triage (ATT) score vs. plasma nucleosome concentration **(D)** Acute Patient Physiology and Laboratory Evaluation (APPLE) score vs. plasma nucleosome concentration. Spearman's correlation coefficients (*r*_s_) with associated *P*-values are displayed in each panel. *P* < 0.05 was considered significant.

Cell-free DNA data are summarized in Figure 3A. Compared to healthy controls in which median plasma cfDNA concentration was 371 ng/mL (306–441), plasma cfDNA concentrations were significantly increased in dogs following trauma 567 ng/mL (404–833), (*P* < 0.0001). There were no associations between cfDNA concentration and lactate, MGCS, ATT, APPLE score, APPLE_fast_ score, SPI2 or SOFA score. Concentrations of cfDNA were positively correlated with shock index, however (*r*_s_ 0.367, *P* = 0.010) (Figure 4A). There was no significant difference in the cfDNA concentrations between survivors 554 ng/mL (395–833) and non-survivors 686 ng/mL (409–1709), (*P* = 0.602), (Figure 3B). Concentrations of cfDNA were not significantly different in dogs with blunt force trauma compared with other mechanisms of injury (*P* = 0.233), or between dogs which were seen by a primary care veterinarian before referral compared with primary walk-in emergencies (*P* = 0.395).

### Nucleosomes

**Figure 5:**
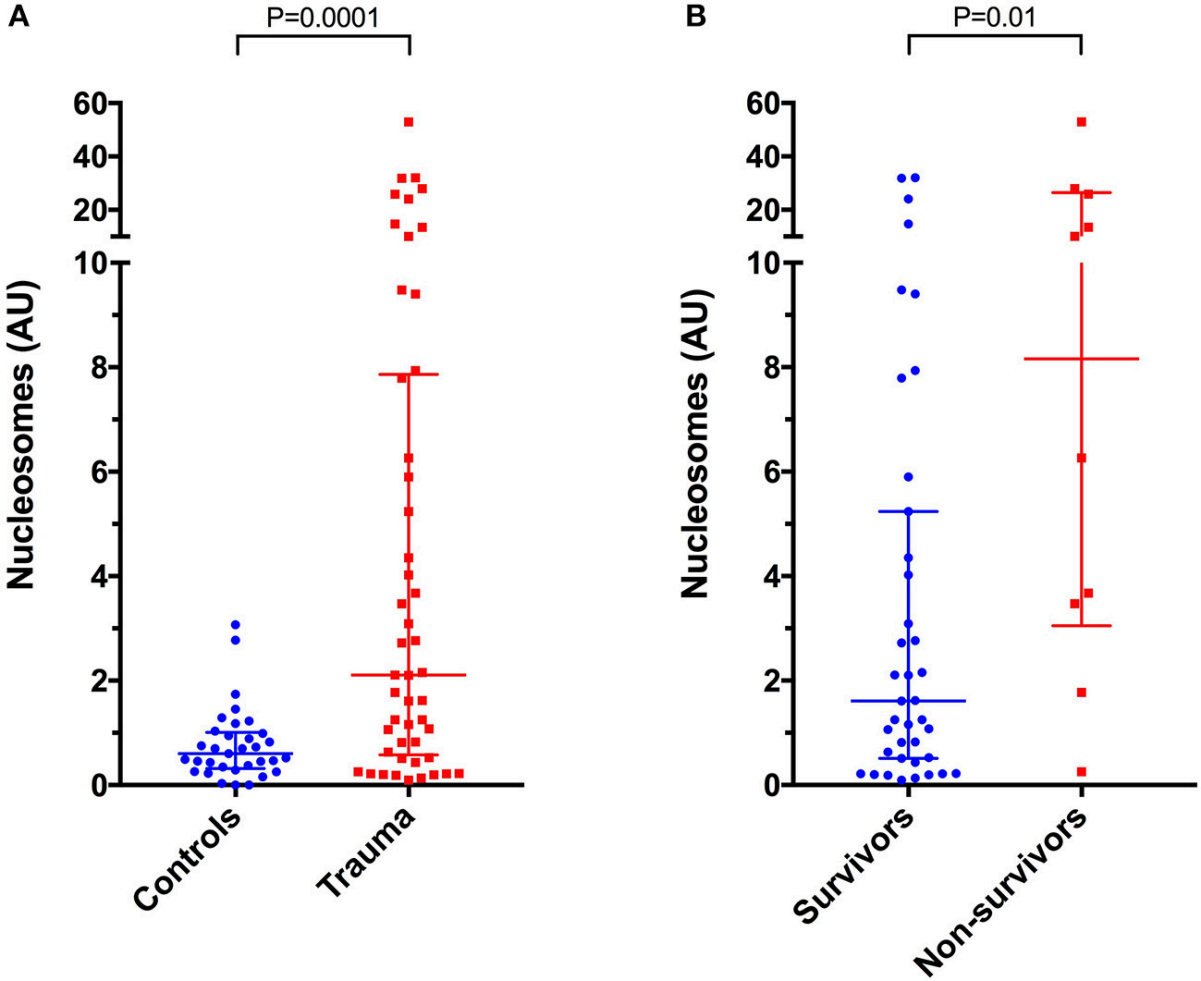
Scatterplots of plasma nucleosome concentrations in arbitrary units (AU) in **(A)** healthy controls (*n* = 33) compared to dogs following moderate-severe trauma (*n* = 49), and in **(B)** survivors (*n* = 39) compared to non-survivors (*n* = 10). The central horizontal line represents the median, and the two error bars represent the interquartile range. Comparisons between the groups were performed using the Mann-Whitney U test. *P* < 0.05 was considered significant.

Compared to healthy controls in which nucleosome median concentration was 0.60 arbitrary units (AU) (0.32–1.01), nucleosome concentrations were significantly higher in dogs following trauma 2.11 AU (0.58–7.87) (*P* = 0.0001) (Figure 5A). Nucleosomes were positively correlated with the shock index (*r*_s_ 0.358, *P* = 0.012), with the APPLE score (*r*_s_ 0.356, *P* = 0.012), and with the ATT score (*r*_s_ 0.327, *P* = 0.022) (Figures 4B–D). There were no associations between nucleosome concentration and lactate, MGCS, APPLE_fast_, SPI2 or SOFA score, however.

**Figure 6:**
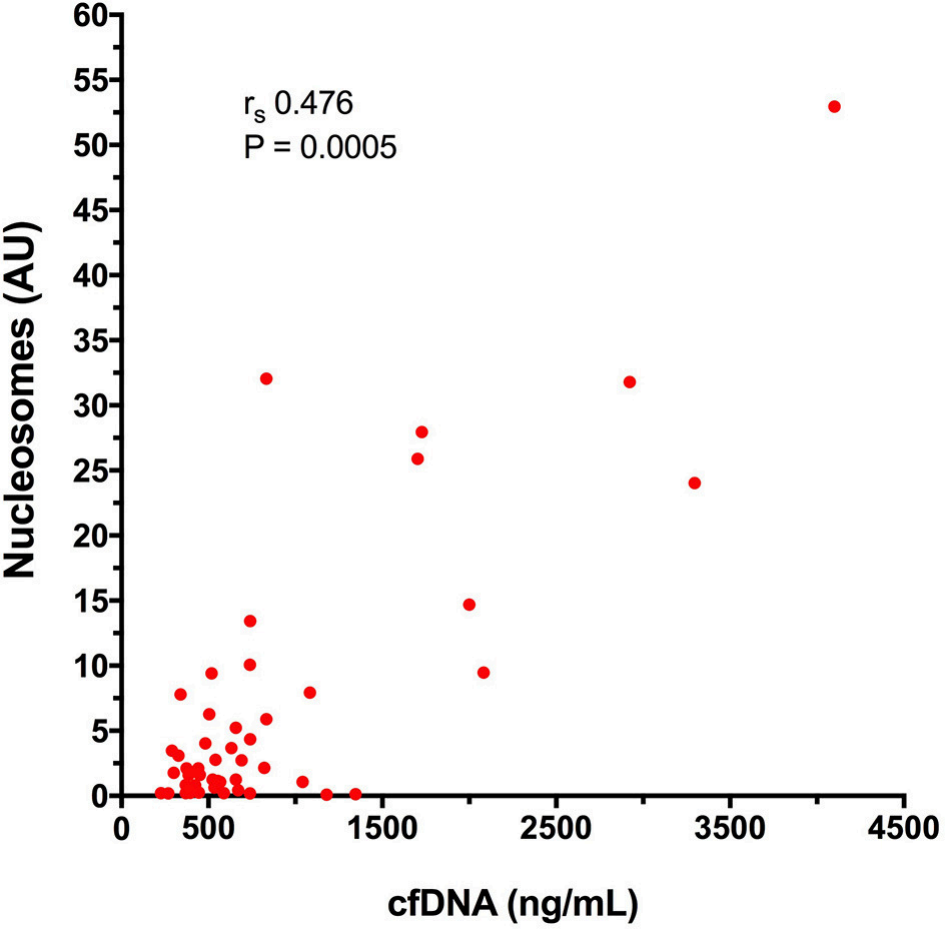
X-Y scatterplot of plasma cell-free DNA (cfDNA) concentration vs. plasma nucleosome concentration in arbitrary units (AU) from 49 dogs after moderate-severe trauma. Spearman's correlation coefficient (*r*_s_) with the associated *P*-value is displayed; *P* < 0.05 was considered significant.

There was a significant positive correlation between cfDNA and nucleosome concentrations (*r*_s_ 0.476, *P* = 0.0005) (Figure 6). Nucleosome concentrations were not significantly different in dogs with blunt force trauma compared with other mechanisms of injury (*P* = 0.087), or between dogs which were seen by a primary care veterinarian before referral compared with primary walk-in emergencies (*P* = 0.094). Median nucleosome concentrations were significantly increased in non-survivors 8.16 AU (3.05–26.40), compared to survivors 1.61 AU (0.51–5.24), (*P* = 0.01) (Figure 5B). Nucleosome concentrations were discriminant for outcome (AUROC 0.762, *P* = 0.011), but logistic regression analysis indicated that nucleosome concentrations were not significantly associated with outcome, independent of illness severity as indicated by the APPLE score.

## Discussion

The present study confirms previous data that following moderate-severe trauma, dogs have significantly increased plasma concentrations of cfDNA (20). The present study also documented a significant increase in plasma nucleosome concentrations in these dogs. The positive correlation documented between these two biomarkers suggests a common mechanism of release for cfDNA and nucleosomes in canine trauma.

The primary aim of the present study was to explore the association between plasma cfDNA concentrations and survival to discharge in dogs following trauma. It was hypothesized that non-survivors would have significantly increased concentrations of cfDNA compared to survivors, however, cfDNA was not demonstrated to be prognostic. This is in contrast to the situation in people where cfDNA is prognostic after multiple trauma or after major surgery (37). It may be that the population of dogs in the present study was not sufficiently injured, since cfDNA appears to be a better prognostic indicator in more severely injured humans (38). In addition, the typical pattern of injury in the present study might have influenced the prognostic utility of cfDNA. In people, high concentrations of cfDNA are more common in penetrating injury than in blunt force trauma (17), whereas the most common mechanism in the present study was blunt force trauma. It is also possible that the lack of association between outcome and cfDNA concentrations was due to the high percentage of non-survivors that were euthanized. Although we attempted to eliminate dogs euthanized for financial reasons, it is possible that some of the dogs euthanized may have survived with continued therapy, confounding the relationship between cfDNA concentrations and outcome. This study was performed at a referral institution, which may have lessened the impact of any financial considerations on client decision-making, but it is not possible to eliminate the potential influence of financial constraints entirely.

It was hypothesized that cfDNA concentrations would positively correlate with illness and injury severity scores, but that was not the case in the cohort of dogs reported here. The shock index was positively correlated with cfDNA concentrations suggesting that release of cfDNA may be associated with the degree of shock following trauma. The simplicity of the shock index calculation and ease of application to assessment of the trauma patients suggested this score may be of value in our patient population. The shock index was calculated using data collected contemporaneous with sample collection for biomarker measurement. Thus, it should reflect the degree of shock present at the time of biomarker measurement irrespective of prior therapy. The other illness severity scores calculated in the present study may capture aspects of the injury type, degree and distribution, or responses to injury that are distinct from the pathophysiologic pathways that lead to cfDNA release. It is also possible that limited injury severity in our population may have made it more difficult to detect an association between cfDNA concentrations and injury severity scores. Although we attempted to focus on moderately to severely injured dogs, the median ATT score was 5 and no dog had a score >13. Additionally, only 15/49 (30.6%) had an APPLE score >30 and our overall mortality rate was 20.4%. The choice of ATT ≥3 as an inclusion criterion was a pragmatic choice. Enrolling patients with greater degrees of injury severity might have increased the usefulness of cfDNA as a prognostic marker. However, that would have limited case recruitment rates and would have made the findings less generalizable given that relatively few canine trauma cases have very high ATT scores (39, 40). Dogs were enrolled in the present study at various amounts of time following their injury and some had already received therapy prior to blood sampling. Although there was no effect of prior therapy on cfDNA concentrations, it is possible that prior therapy might have affected the correlations between cfDNA and illness severity scores.

Of the illness and injury severity scores, only the APPLE score was significantly associated with survival to hospital discharge. Our analyses of these associations were exploratory only and were not intended to validate the scores, but rather to provide information about the level of injury and resulting physiologic derangements that resulted from the trauma. These analyses also provided context for our investigation of two novel biomarkers and enabled illness severity to be accounted for when correlating biomarker concentrations with outcome. In our small population most scores were not significantly associated with survival, while in contrast, in a recent study employing a large canine trauma registry both ATT and MCGS are associated with non-survival in dogs following injury (41). This suggests our small sample size was limiting. Although the APPLE score was associated with survival in the present study, this association is confounded by the number of patients that were euthanized and the potential that a clinician's knowledge of illness severity may have influenced decision-making and client discussions. The only point-of-care test that was associated with outcome in the present study was lactate, but there was significant overlap between the values of survivors and non-survivors that might limit the prognostic utility of a single time point lactate measurement.

The processes that lead to the release of cfDNA may include necrosis, apoptosis and neutrophil extracellular trap formation. Increased plasma cfDNA concentrations may be caused by increased release of cfDNA and/or decreased clearance. The mechanisms of metabolism and clearance of cfDNA from plasma are not fully understood, but likely involve the liver, spleen and kidneys (33, 42). Since the metabolism and clearance of cfDNA are incompletely understood, it is possible that some patients had alterations in cfDNA clearance that affected measured concentrations without altering illness severity scores. A study of cfDNA in dogs with sepsis suggests that this biomarker may be useful for the early identification of patients with bacteremia (28), a finding that may derive from increased intravascular NETosis in dogs with bacteremia. The lack of association between injury severity and cfDNA concentrations in the present study might suggest that NETosis does not play a prominent role in dogs following moderate-severe trauma. In contrast to the data on cfDNA, the present study determined that plasma nucleosome concentrations were significantly increased in non-survivors compared to survivors. Nucleosomes were also positively correlated with several illness severity scores, suggesting that plasma nucleosome concentrations may provide better biologic insight into the pathophysiologic consequences of trauma in dogs.

We recognize our study has limitations. Some of our patients may have had comorbidities that affected cfDNA concentrations distinct from their trauma processes, although this is less likely given the overall young median age of the trauma population. Cell-free DNA and nucleosome concentrations were measured only at enrollment. Dogs were presented at different times during the course of disease and since the kinetics of cfDNA and nucleosome release in trauma are unknown, but are likely dynamic, this may have affected measured concentrations in an unpredictable manner. Serial evaluation of these markers over time may enhance the usefulness and better define the role of those biomarkers in the assessment of prognosis after trauma. The nucleosome ELISA we employed was designed for assessment of apoptosis in cultured cells and hence does not have a reference standard to enable absolute concentrations to be established. To circumvent this, we scaled the concentrations of plasma nucleosomes against pooled plasma from healthy blood donor dogs. It was assumed that this pool of dogs had normal nucleosome concentrations, but precludes reporting an actual value for the nucleosome concentrations in the dogs following trauma. Nucleosome concentrations were analyzed in 2 batches within 15 months of sample collection. A study on the effect of long-term sample storage on human serum nucleosome measurements suggests that some minor diminution in concentrations occurs over a 5-year period at −70°, although it is not known if the same is true in dogs (43). Some data were missing which necessitated median imputation to enable calculation of illness-severity scores. There is little agreement in the literature on the best way to handle missing data, and all methods have their shortcomings (44). Median imputation was chosen because it was straightforward to achieve and was consistent with the methods used to derive the APPLE score. Median imputation is comparable to other simple methods of imputation and may be preferable to case-wise elimination (45).

In summary, this study demonstrates that cfDNA and nucleosome concentrations are increased in dogs following moderate-severe injury and that nucleosome concentrations are greater in dogs that did not survive to hospital discharge, compared to survivors. Concentrations of cfDNA and nucleosomes are positively correlated with shock index and nucleosome concentrations are positively correlated with illness and injury severity. Further studies evaluating the kinetics of nucleosome concentrations over time in trauma patients appear warranted to determine the optimal strategies for use of this novel biomarker.

## Ethics statement

All samples analyzed in this study were collected from dogs managed at the institution veterinary teaching hospital (Cornell University, Ithaca, NY) as part of studies approved by the local Institutional Animal Care and Use Committee (IACUC), and undertaken under written informed client consent (Cornell IACUC 2014-0053). Healthy privately-owned dogs were enrolled as controls with local Institutional Animal Care and Use Committees approval and written informed client consent (Cornell IACUC 2014-0052).

## Author contributions

RG designed the study, collected and analyzed data, and co-wrote the manuscript; J-AL assisted with study design, collected and analyzed data, and co-wrote the manuscript. Both authors contributed to, read and approved the final manuscript.

### Conflict of interest statement

The authors declare that the research was conducted in the absence of any commercial or financial relationships that could be construed as a potential conflict of interest.

## Abbreviations

APPLE: acute patient physiology and laboratory evaluation score
ATT: animal trauma triage score
cfDNA: cell-free DNA
NETs: neutrophil-extracellular traps
NETosis: neutrophil extracellular trap formation
SOFA: sequential organ failure assessment
SPI2: survival prediction index-2.
